# Cyclodextrin Complex Formation with Water-Soluble Drugs: Conclusions from Isothermal Titration Calorimetry and Molecular Modeling

**DOI:** 10.1208/s12249-021-02040-8

**Published:** 2021-08-31

**Authors:** Karim S. Shalaby, Muhammad I. Ismail, Alf Lamprecht

**Affiliations:** 1 Department of Pharmaceutics, Institute of Pharmacy, University of Bonn, Gerhard-Domagk-Str. 3, 53121 Bonn, Germany; 2 Department of Pharmaceutics and Industrial Pharmacy, Faculty of Pharmacy, Ain Shams University, Cairo, Egypt; 3 Department of Pharmaceutical Chemistry, Faculty of Pharmacy, The British University in Egypt, Cairo, Egypt; 4 PEPITE EA4267, University of Burgundy / Franche-Comté, Besançon, France

**Keywords:** isothermal titration calorimetry, molecular modeling, cyclodextrin, water-soluble drugs, tramadol

## Abstract

Cyclodextrin (CD) complexes are frequently used for enhancing the solubility or absorption of poorly water-soluble drugs. On the contrary, little is known about their complex formation with water-soluble drugs. Here, we have studied the interaction between 2-hydroxypropyl β-CD (HPβCD) and three water-soluble drugs, namely naloxone (NX), oxycodone (OC), and tramadol (TR), by isothermal titration calorimetry (ITC) combined with molecular modeling in view of the potential impact on drug release. The results showed that the complex formation of HPβCD with all three drugs occurs spontaneously. The complexes formed with NX and OC were found to be 2NX:1HPβCD and 3OC:2HPβCD, respectively. TR was found to form 2 complexes with HPβCD; of 1:2 and 1:1 complexation ratios. The binding of HPβCD to NX was greater than to OC due to the higher hydrophobicity of the structure of the former. Moreover, the binding affinity of HPβCD to TR was higher than to OC, which indicated the effect of the higher flexibility of the guest in increasing the binding affinity. *In vitro* drug release experiments from the various complexes revealed a significant impact of the stoichiometry of the complex on the release profiles. Accordingly, the co-administration of cyclodextrins with water-soluble drugs should be closely monitored, as it may result in unintentional complex formation that can potentially impact the drugs’ gastrointestinal absorption.

**Supplementary Information:**

The online version contains supplementary material available at 10.1208/s12249-021-02040-8.

## INTRODUCTION

Cyclodextrins (CD) are cyclic macromolecules composed of different numbers of glucose units linked by α-1,4-linkage to form a ring structure. Natural CD are composed of 6, 7, or 8 glucose units and called α-, β-, and γ-CD, respectively. Due to the limited solubility of unsubstituted natural CD, more soluble derivatives, e.g., 2-hydroxypropyl β-CD (HPβCD) and sulfobutylether β-CD (SBEβCD), have been developed (1,2). Their idealized model shape resembles a truncated cone with a lipophilic central cavity and a hydrophilic outer surface. By virtue of hosting hydrophobic drugs in the central cavity, CD increase the solubility of poorly water-soluble molecules (3–7).

As the drug-CD complex has physicochemical properties different to that of the drug itself, they facilitate the delivery of drugs with poor water solubility or chemical instability, increasing thereby the therapeutic efficiency (6). On the other hand, some CD, particularly those of more hydrophobic nature, have been used to modify or delay the release of some water-soluble drugs, e.g., captopril and metoprolol, to prolong their pharmacological effect (8–10). Accordingly, CD are frequently used as excipients in the pharmaceutical industry (11), mainly for the purpose of enhancing the solubility of water-insoluble drugs. However, limited information is available regarding the binding of CD with water-soluble drugs, though this could be of great importance in cases where CD and water-soluble actives are concomitantly administered. In such cases, potential interactions therebetween may impact the drug permeability through absorption barriers including but not limited to the gastrointestinal tract.

Various factors have been shown to impact the binding between CD and various drugs. Some are related to the CD molecule itself, including the size of the cavity (13) and the number of water molecules therein (12). Others are related to the guest molecule structure such as molecular length, composition, atomic distribution, and the presence of electronically withdrawing substituents (13,14). Within this context, diverse analytical methods have been applied to identify the thermodynamic profile of CD-drug complexation reaction, e.g., ^1^H-NMR, differential scanning calorimetry (DSC), UV spectroscopy, fluorimetry, isothermal titration calorimetry (ITC), and molecular modeling (15,16).

Among these, ITC has been extensively used for the development of a complete and precise thermodynamic profile of many binding interactions, including the binding between CDs and various lipophilic drugs, where the binding energy was large enough to be measured (17–19). However, the potential of the ITC to analyze less stable binding interaction with low binding energies, e.g., CDs and water-soluble compounds, is yet to be evaluated. Moreover, the curves produced by ITC are sometimes rather complex and cannot be easily interpreted (20). Therefore, the combination of ITC experimental techniques with theoretical perspectives, e.g., molecular modeling, is helpful to interpret ITC complex curves and for developing a three-dimensional illustration of the most stable inclusion complexes (19).

In this study, a combination of ITC and molecular modeling was used to explore and interpret the interaction between HPβCD and three water-soluble opioid drugs: naloxone hydrochloride (NX), oxycodone hydrochloride (OC), and tramadol hydrochloride (TR). It was demonstrated that, even after dissolution, the unintended interactions between water-soluble drugs and CD can lead to complex formation. Since CD cannot pass the gastrointestinal barrier, the interaction of such drugs with these molecules might lead to a delay in their absorption in the gastrointestinal tract. Moreover, as the model drugs selected in the current study are structurally related but differ in hydrophobicity and rigidity in their structures (Fig. S1), it was possible to determine the effect of the hydrophobicity and the rigidity of the hydrophilic guest molecule on their affinity to HPβCD.

## MATERIALS

Hydroxypropyl β-cyclodextrin (HPβCD) was purchased from TCI Deutschland GmbH (Eschborn, Germany). Naloxone HCl, oxycodone HCl, and tramadol HCl were purchased from Sigma-Aldrich (St. Louis, USA). Regenerated cellulose membrane (molecular weight cutoff = 1000 Da) was purchased from VWR (Darmstadt, Germany). All other chemicals were of analytical grade.

## METHODS

### Isothermal Titration Calorimetry (ITC)

Aqueous solutions of NX (171.7 mM), OC (257.4 mM), TR (257.4 mM), and HPβCD (10 mM) were cooled and degassed using the Themovac-2 sample degasser and thermostat. ITC measurements were carried out at 298.15 K (MicroCal VP-ITC® microcalorimeter, Northampton, USA). The degassed drug solution was filled into the injection needle of the device (299 μL) and HPβCD solution was filled into the cell (1.43 mL). Titration parameters were set to 58 injections of 5 μL of the titrant each for 6 s, and the interval between injections was set at 200 s and the stirring rate at 310 rpm. The binding isotherm was developed by detection of the heat resulted from each injection. The overall heat was thus measured and used to plot the binding isotherm.

The resulted binding isotherms were analyzed using the fit model of the Origin 5.0® software with the Levenberg-Marquardt algorithm. All data analyses were performed after subtraction of the heat of dilution of both the drug and the HPβCD in each case. To this end, the heat resulted from the injection of the drug into water and the injection of water into the HPβCD solution was subtracted from that obtained for each injection. Based on the analysis and fitting of the data, the value of stoichiometry (N), the binding constant (K), the change in enthalpy due to binding (ΔH), the change in Gibb’s free energy (ΔG), and the change in entropy (ΔS) were developed. ΔH and ΔS were used to determine the type of binding, whereas ΔG was used to determine the spontaneity of the binding.

### Molecular Modeling of Drugs with HPβCD

The crystal structure of β-CD was downloaded from the Cambridge Structural Database (CSD reference code (BUVSEQ02) (21), and modified using Avogadro software to obtain the molecular structure of 2-HPβCD at C6 (22,23). Generalized Amber force field (GAFF) was used to minimize the resulting structure (24). Ligand structures were constructed using MarvinSketch (25) and minimized using MMFF94s force field (26). All minimization runs were implemented via Open Babel software with 10,000 steps using the steepest descent algorithm (27). Molecular docking was carried out using AutoDock Vina software (28) implemented through PyRx GUI (29). All figures were generated using UCSF Chimera (30). Through these methods, the binding of two molecules of HPβCD together to form a dimer was simulated and compared to the binding of HPβCD to each of the different drug molecules. The formation of complexes of different stoichiometries was also studied to determine the most stable complex between HPβCD and each drug.

### The Release Profile of NX, OC, and TR from Complexes

The dissociation and release of NX, OC, and TR from complexes were analyzed by means of vertical Franz cells (SES GmbH, Bechenheim, Germany) with a regenerated cellulose membrane (molecular weight cutoff = 1000 Da) and an acceptor compartment filled with 8 mL of a buffered solution of pH 2. Solutions of NX, OC, and TR, or their complexes with HPβCD (NX-HPβCD, OC-HPβCD, or TR-HPβCD) were added to the donor compartment. As the complexes are spontaneously formed in water, the complexes’ solutions were prepared by mixing of the pure drug solution with solution of HPβCD. The mixture was then maintained for 20 min to achieve an equilibrium. A total of 300 μL of NX or its complex with HPβCD, or 200 μL of OC, TR, or their resultant complexes, was added to the donor compartment. The concentrations of NX, OC, and TR in all solutions were 320 mM, 360 mM, and 12 mM, respectively. In all cases, the experiment was carried out under a controlled temperature of 37°C (JULABO GmbH, Seelbach, Germany) and in triplicate. At predetermined time points, samples (500 μL) each were withdrawn from each cell and replaced by a fresh buffer.

The samples were analyzed using HPLC (Agilent Technologies, Waldbronn, Germany) by modification of previously reported methods (31–33). Briefly, NX HCl was quantified using a C8 column LiChrospher 60 RP Select B-5 μ (CS-Chromatographie, Merck) at 60°C. The mobile phase was composed of buffer (pH 2) and methanol (85:15). The flow rate and injection volume were 1 mL min^−1^ and 10 μL, respectively. The detection wavelength was 230 nm. Limit of detection (LOD) and limit of quantification (LOQ) were calculated as 330.38 μg/mL and 1002.53 μg/mL, respectively. The quantification of OC HCl was performed analogously, using a C8 column LiChrospher 60 RP Select B-5 μ (CS-Chromatographie, Merck), with the same mobile phase, and at the same flow rate and temperature. Peaks were detected at 230 nm. LOD was 15.56 μg/mL and LOQ was 47.14 μg/mL.

TR HCl, on the other hand, was quantified using a LiChrospher® RP-18 HPLC Column (5 μm particle size, L × I.D. 15 cm × 3.2 mm) (CS-Chromatographie, Merck) at room temperature. The mobile phase consisted of phosphate buffer (pH 6.0):methanol (40:60). The flow rate was set to 1.0 mL min^−1^ and the detection wavelength was 270 nm. Under such conditions, the LOD and LOQ were equal to 22.62 μg/mL and 68.54 μg/mL, respectively.

## RESULTS

### NX and HPβCD

**Fig. 1:**
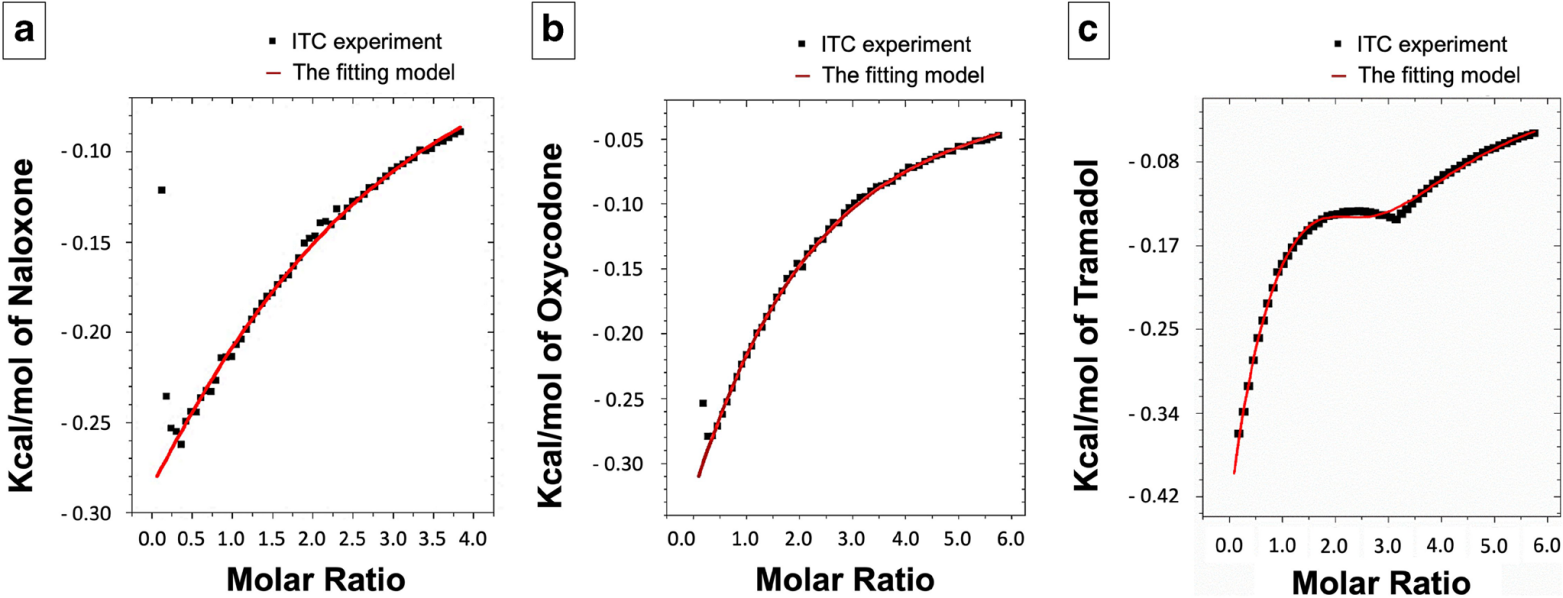
The binding isotherm of the binding of **a** NX, **b** OC, and **c** TR with HPβCD after subtraction of the dilution heat of the drugs and HPβCD then fitting

Following the subtraction of the dilution heat of both compounds, the binding isotherm originating from the titration of NX (171.7 mM) and HPβCD (10 mM) comprises three phases (Fig. 1a). In the first phase, small but increasing amounts of heat evolved (exothermic peaks), until a maximum was reached. Afterward, the amount of heat produced tended to decrease steadily giving the curve a linear appearance with a nearly constant slope (the second phase). In the third phase, the heat produced tended to be constant and the curve started to form a plateau. This curve resembles the normal sigmoid binding isotherms except in the first phase, in which there are small, yet increasing in size, exothermic peaks. The curve was fitted to the one set of identical site model after omitting the first phase (Fig. 1) and the binding parameters were developed (Table I).

**Table I:** The Thermodynamic Parameters for the Binding of Naloxone HCl, Oxycodone HCl, and Tramadol HCl with 2-Hydroxypropyl β-Cyclodextrin After Subtraction of the Dilution Heat of the Drugs and 2-Hydroxypropyl β-Cyclodextrin then Fitting

Parameter	Naloxone	Oxycodone	Tramadol	Unit
N	2.036 ± 0.02	1.46 ± 0.08		
ΔH_1_	− 523.8 ± 14.42	− 759.50 ± 28.99	− 443.97 ± 26.08	kcal/mol
K_1_	56.78 ± 2.01	48.50 ± 2.14	814.17 ± 89.18	M^-1^
TΔS_1_	1868.80 ± 35.42	1539.80 ± 2.74	3524.13 ± 60.88	kcal/Mol
ΔG_1_	− 2392.6 ± 20.99	− 2299.30 ± 26.25	− 3968.10 ± 62.68	kcal/mol
K_2_			352.73 ± 16.02	M^-1^
ΔH_2_			129.74 ± 39.87	kcal/mol
TΔS_2_			3604.63 ± 16.60	kcal/mol
ΔG_2_			− 3474.90 ± 27.07	kcal/mol
K_3_			60.17 ± 6.07	M^-1^
ΔH_3_			− 810.43 ± 20.11	kcal/mol
TΔS_3_			1614.88 ± 41.34	kcal/mol
ΔG_3_			− 2425.31 ± 61.18	kcal/mol

Based on the calculated values (Table I), the stoichiometry of the binding of NX to HPβCD is 2:1. The values of ΔH and ΔG are negative and the value of enthalpy is smaller than that of entropy (|ΔH|<|TΔS|).

**Fig. 2:**
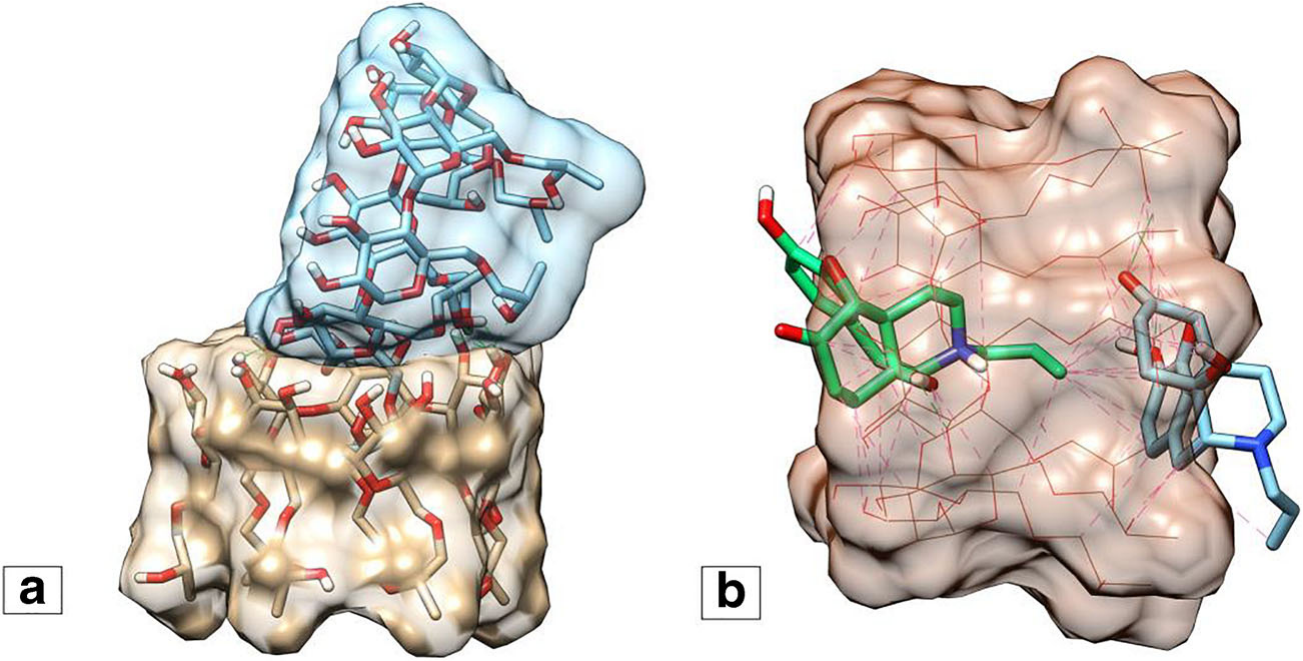
**a** HPβCD dimer predicted by molecular docking. **b** NX-HPβCD complex with ratio 2:1 predicted by molecular docking. The two NX molecules are depicted in green (front) and blue (back). The hydrogen bonds are in green dashed lines and the hydrophobic contacts are in magenta

The docking score (ΔG) related to the docking of NX into HPβCD (− 7 kcal/mol) is more negative than representing the docking of one HPβCD molecule into another (− 4.0 kcal/mol). Docking another molecule of NX into NX-HPβCD receptor molecule gives a binding energy of − 5.0 kcal/mol which indicates a favorable binding of a second NX molecule to the NX-HPβCD complex (Fig. 2).

### OC and HPβCD

Similar to the case of NX, the binding isotherm originating from the titration of OC (257.4 mM) and HPβCD (10 mM) (following the subtraction of the dilution heat of OC and HPβCD) is triphasic. Hence, the net heat evolved in the first phase was also omitted before fitting of the curve to the one set of identical site model (Fig. 1b), which enabled calculating the values of the binding parameters (N, K, ΔH, and ΔS) developed. From these values, TΔS and ΔG were calculated (Table I). The stoichiometry of the binding of OC to HPβCD is about 1.5 which suggests that a 1.5:1 ratio is energetically suitable; e.g., 3 molecules of OC bind to 2 molecules of HPβCD. As in the case of NX, the values of ΔH and ΔG are negative, and the value of enthalpy is smaller than that of entropy (|ΔH|<|TΔS|).

**Fig. 3:**
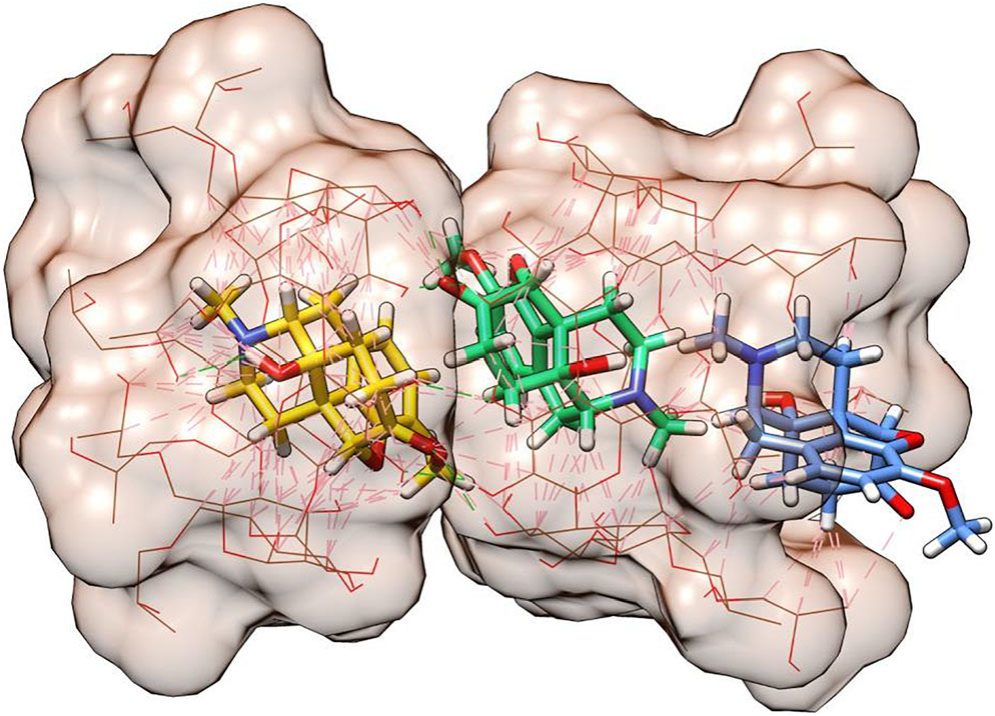
Complex of 3OC:2HPβCD predicted by stepwise docking. Initial HPβCD molecule (khaki) on the right, used as a receptor, and the docked HPβCD (khaki) on the left. First, second, and third OC docked molecules are shown as blue (right), green (middle), and gold (left) sticks, respectively. The hydrogen bonds are as green dashed lines, and the hydrophobic contacts are in magenta

OC was docked into HPβCD to determine its binding affinity, where the calculated docking score of − 6.9 kcal/mol supported the experimentally performed isotherm. To determine the ability of OC to form complexes with HPβCD, a stepwise docking approach was applied. This was performed by docking different molecules of OC and HPβCD to previously formed complex in each step, e.g., a second molecule of OC to the OC-HPβCD complex, a third OC molecule to the OC-OC-HPβCD complex, and finally docking HPβCD to the OC-OC-OC-HPβCD complex, and so on (Fig. 3). The calculated docking score in kcal/mol was developed in each binding step (Table II).

**Table II:** Binding Affinities (kcal/mol) for the Building up Process of OC-HPβCD 3:2 Complex Through Stepwise Docking

Docking step	Ligand	Receptor	Docking score (kcal/mol)
1	OC	HPβCD	-6.9
2	OC	OC-HPβCD complex	-5.9
3	OC	OC-OC-HPβCD complex	-5.1
4	HPβCD	OC-OC-OC-HPβCD complex	-3.9

*OC* oxycodone HCl, *HPβCD* 2-hydroxypropyl β-cyclodextrin

### TR and HPβCD

After the subtraction of the dilution heat of TR and HPβCD, the binding isotherm originating from the titration of the TR (257.4 mM) and HPβCD (10 mM) comprises 4 phases (Fig. 1c). The first phase consists of large, but getting smaller, exothermic peaks. The second phase shows exothermic peaks, which increase in size, and is followed by a phase of exothermic peaks, which get smaller. Finally, the curve starts to form a plateau-like behavior, which represents the fourth phase. The whole curve was fitted to the three sequential binding site model of the Origin 5.0® software as shown in Fig. 1c (the red curve), and the values of fitting parameters (Kn, ΔHn, and ΔSn) for every interaction and, subsequently, the values of ΔSn and ΔGn were calculated (Table I). It should be noted that n indicates the first, the second, or the third interaction.

To determine the stoichiometry of the binding of TR with HPβCD, the data obtained from the first phase only was fitted to the one set of identical site model and the resultant stoichiometry indicated that the complex was composed of 2 molecules of HPβCD and one molecule of TR. On the contrary, when the third and last phase of the curve was fitted to the one set of identical site model, the resultant stoichiometry indicated the presence of a 1:1 complex molecule (data not shown).

**Fig. 4:**
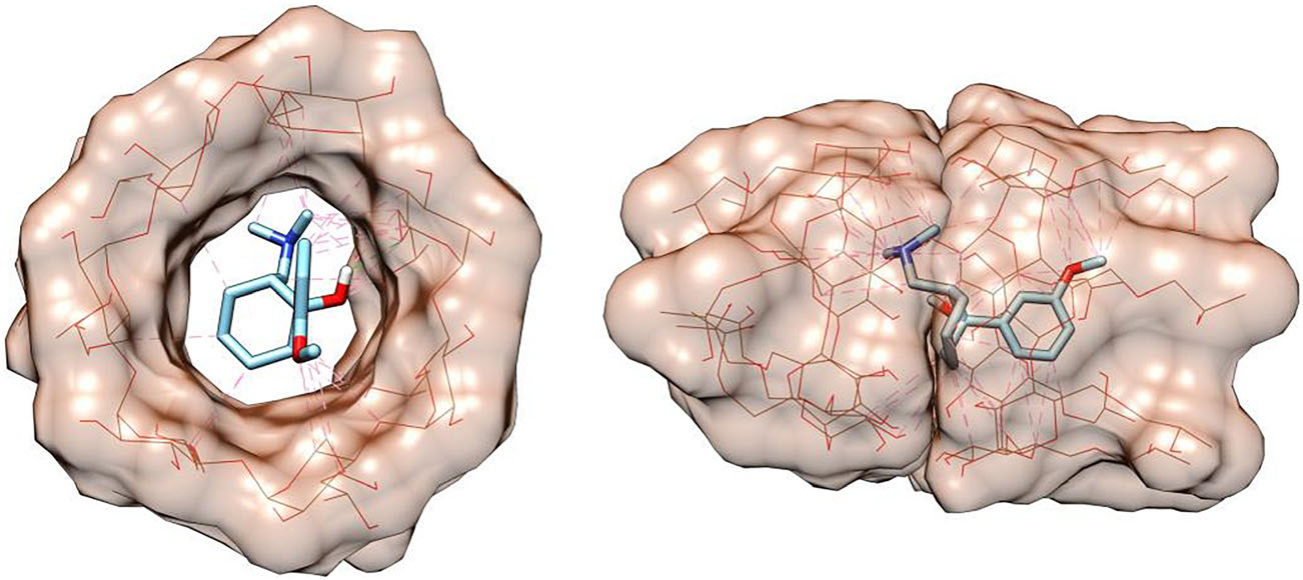
3D view of TR (blue sticks) docked in a monomer (left) and a dimer (right) of HPβCD (brown wire). The hydrogen bonds are as green dashed lines, and the hydrophobic contacts are in magenta

Here again, computational calculation of binding affinity (ΔG) was carried out using molecular docking, and the predicted binding affinity in kcal/mol of each docking complex is shown in Table III. Additionally, the predicted TR-HPβCD complexes are shown in Fig. 4.

**Table III:** Docking Experiments Showing the Receptor and the Ligand in Each Docked Complex, and the Resulting Binding Affinity in kcal/mol

Docked complex	Ligand	Receptor	Docking score (kcal/mol)
Tramadol-tramadol	Tramadol	Tramadol	− 2.9
HPβCD-HPβCD	HPβCD	HPβCD	− 4.0
Tramadol-HPβCD-HPβCD	Tramadol	HPβCD-HPβCD complex	− 8.0
HPβCD-tramadol-HPβCD	HPβCD	Tramadol-HPβCD complex	− 3.1
Tramadol-HPβCD	Tramadol	HPβCD	− 6.0

### The Release Profile of NX, OC, and TR from Complexes

**Fig. 5:**
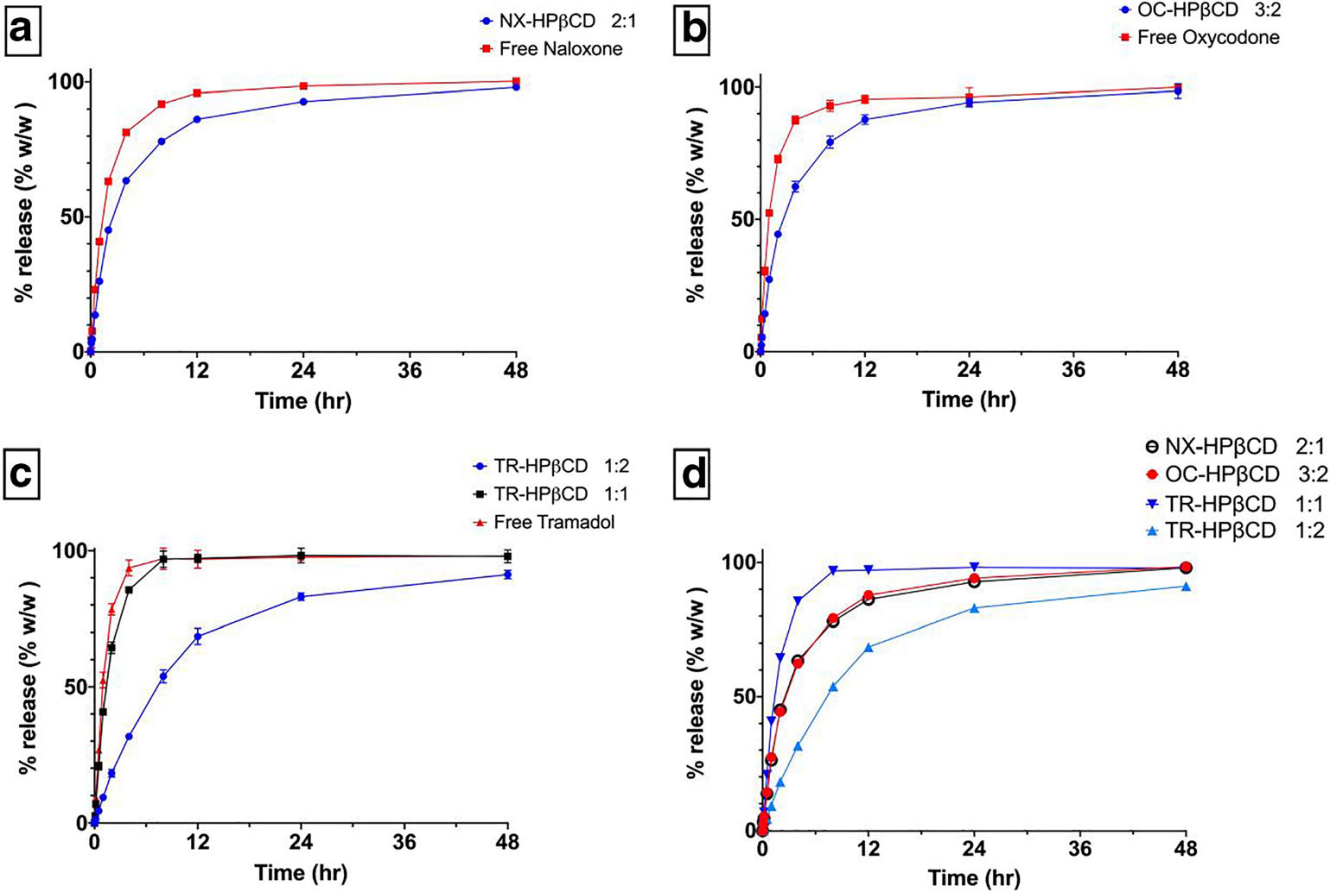
The release profile of NX **a**, OC **b**, and TR **c** and their complexes with HPβCD in a buffer of pH2. **d** The release profile of all complexes

The release of the drugs from their complexes with HPβCD into the acceptor compartment showed a more delayed profile than the release of the corresponding free drugs (Fig. 5a–c). Comparing the release profile of complexes that have different stoichiometry, it was found that the delay in drug release is dependent on complex stoichiometry (Fig. 5d).

## DISCUSSION

ITC analyses of the NX-HPβCD complexes yielded a triphasic curve (Fig. 1a). In this case, rather than high nearly equal exothermic peaks expected during the first phase of the normal isotherm (sigmoid curve), small, but becoming larger, exothermal peaks are observed. A potential explanation for this phenomenon is the presence of HPβCD as dimers in the solution, which is in line with an earlier report (34). Consequently, the observed peaks in the first phase are related to the dissociation of HPβCD dimers (endothermic) and the formation of NX-HPβCD complex (exothermic). This explains the initial small, but increasing in size, exothermic peaks which continued until all HPβCD dimers are separated into single molecules. Thereafter, NX binds to HPβCD monomers resulting in a decreasing pattern of peaks (phase 2), as the number of free HPβCD molecules decreases. Once a 2:1 binding is fully achieved, further addition of NX cannot result in any subsequent binding, accounting for the observed reduced steepness of the slope. Since the net heat developed in the first phase is not purely related to the binding between HPβCD and NX, it was omitted before fitting the curve to the single-site model and the subsequent calculation of the binding parameters. The score calculated for the docking of NX into HPβCD (− 7.0 kcal/mol) is more negative than that of HPβCD molecule docked into another HPβCD molecule (− 4.0 kcal/mol), which supports the hypothesis that NX displaces one molecule of HPβCD in HPβCD dimer to make a more stable complex with HPβCD. The docking of another molecule of NX into NX-HPβCD is favored as indicated by the favorable binding energy (− 5.0 kcal/mol). Besides, the sum of these energies together was more favorable than the binding energy of two HPβCD molecules to form a dimer which supports the experimental results of NX forming a complex with HPβCD in a 2:1 ratio.

We also aimed to investigate the effect of drug hydrophobicity on its complex formation with HPβCD. To this end, we included OC within the experiments, as it is structurally similar to NX but is less hydrophobic in nature. ITC and molecular docking results show that 3 molecules of OC bind to 2 molecules of HPβCD. The lower stoichiometry value indicates a lower affinity of HPβCD to OC when compared to NX. This is also supported by the lower value of the binding constant (K) and the absolute value of Gibb’s free energy (ΔG) for OC than for NX.

Another goal within the context of the current study was to investigate the effect of the difference in structural rigidity of the drug upon its complex formation with HPβCD. Accordingly, we also included TR within the experiments, as it possesses structural similarity with OC but less rigid due to a lower number of rings in its structure. The ITC experiment yielded a complex interaction curve composed of 4 phases (Fig. 1c). We hypothesized that phase 1 involves the binding of one TR molecule with two molecules of HPβCD to form the 1:2 complex as previously reported (16) and as detected by fitting this phase to a single binding site model (data not shown). Though this binding has probably resulted in the production of a high amount of heat, a part of this heat has been seemingly absorbed for dissociation of TR dimers as well as HPβCD dimers. Hence, the net exothermic peaks are smaller in value than they should be. In phase 2, further addition of TR up to a definite concentration leads to the dissociation of one molecule of HPβCD from the complex 1TR:2HPβCD (endothermic), followed by the binding of the released molecule of HPβCD with another molecule of TR (exothermic). Since the heat absorbed for complex dissociation is lower than the heat evolved in new complex formation, the net result has been manifested as exothermic peaks. These peaks become larger as the number of 1TR:2HPβCD molecules continuously decreased. This phase continues until all 1TR:2HPβCD complex molecules are dissociated. After that, new molecules of TR added in phase 3 continue to interact with the remaining free molecule of HPβCD to form the 1:1 complex. This accounts for exothermic peaks, which become smaller due to the decreasing amount of free HPβCD remaining in the cell. Lastly, in phase 4, addition of TR led to no further interaction, as all HPβCD molecules have been complexed, giving rise to the curve having a near plateau shape.

Computational calculation of the binding affinity between TR and HPβCD was used to interpret the ITC behavior of TR-HPβCD complexation. Based on these investigations, we hypothesize that the high exothermic interactions observed in phase 1 are the result of the binding of one molecule of TR to two molecules of HPβCD with predicted ΔG = − 8.0 kcal/mol. The heat evolved from this exothermic interaction exceeds the endothermic energy required to dissociate one TR dimer and one HPβCD dimer (+2.9 and +4.0 kcal/mol, respectively). Therefore, endothermic and exothermic energies have resulted in a net ΔG = |8.0| − |2.9| − |4.0| = |1.1| kcal/mol, which means that the net result is an exotherm but less in value than the normal exotherm expected from the formation of the 1TR:2HPβCD complex. In phase 2, the combined endotherm required to dissociate one TR dimer (+2.9 kcal/mol) and one HPβCD molecule from 1TR:2HPβCD (+3.1 kcal/mol) is comparable to the exotherm released due to the formation of the 1TR:1HPβCD complex (− 6.0 kcal/mol), which gives phase 2 a nearly plateau shape. In phase 3, the exotherm released due to the formation of the 1TR:1HPβCD complex is dominant and endows phase 3 its exothermic behavior.

Both the binding affinity and docking score of TR to 2 molecules of HPβCD are higher than to 1 molecule of HPβCD. This could be attributed to the higher number of hydrophobic interactions between one molecule of TR and 2 molecules of HPβCD, when compared to that between one molecule of TR and one molecule of HPβCD. During the formation of the 1:2 complex, the TR molecule has the ability to bind to the most favorable binding positions in 2 molecules of HPβCD, resulting in a maximum number of hydrophobic interactions. Therefore, it has high binding affinity and docking score. However, the limited number of hydrophobic interaction sites in 1 molecule of HPβCD during 1:1 complex formation accounts for the lower binding affinity and docking score.

Based on the combination of ITC and molecular modeling, it can be detected that TR forms two types of complexes with HPβCD (1:2 and 1:1). This explains the contradicting results obtained from the two previously performed studies (15,16). In the first study, the complexation of TR with β-CD was examined using ^1^H-NMR and UV and fluorescence spectroscopy, where the inclusion of TR inside the cavity of β-CD did not affect the absorbance values, but increased the intensity of its fluorescence emission in highly concentrated TR solutions only. On the other hand, it did not affect the fluorescence emission from solutions of lower concentrations that do not normally suffer from a reduction in fluorescence due to self-quenching (15). The self-quenching behavior of TR reported in that study may support our assumption that TR exists as dimers in concentrated solutions. The ratio for the complex formation that was concluded from that study was 1:1. This was contradicting to the results of a second study, in which the inclusion complex of β-CD with TR was investigated by ^1^HNMR, FT-IR, DSC, and scanning electron microscope (SEM), where the complex was reported to be of 2 β-CD and 1 TR molecules (16).

In cases of all three hydrophilic drugs investigated herein, the binding is due to the hydrophobic interaction with the hydrophobic cavity of HPβCD. This is accompanied by the release of a water molecule from the cyclodextrin cavity, which is represented by the high entropy and lower enthalpy of interactions as well as the high number of hydrophobic contacts between HPβCD and the drugs (magenta dashed lines in 3D figures of the complexes). Accordingly, the presence of more hydrophobic groups in high numbers increases the affinity of the water-soluble guest molecule to the CD molecule. Additionally, higher flexibility of the guest molecule enhances its ability to properly position its hydrophobic groups into the cavity, strengthening thereby the hydrophobic interactions. This is confirmed by the higher binding constant (ΔG) and the binding affinity (K) of TR to HPβCD compared with the other two drugs, which signifies a lower structural rigidity to increase its affinity to form complex with CD.

It should be also noted that all the binding interactions studied here were exothermic as indicated by the negative values of ΔH. Surprisingly, the values of ΔG were also negative demonstrating that the binding interactions of CD, even with water-soluble molecules, are spontaneous and favored. This means that the presence of CD together with these water-soluble drugs in the same solution will result in spontaneous complex formation without the need for special preparation procedures. This finding has various implications, as it can impact drug formulations in many different ways. For instance, drug availability and permeation, as demonstrated here with *in vitro* drug release tests, can be strongly modified. As observed in this case, the diffusion of NX, OC, and TR through a dialysis membrane was generally slower when the drug was in a complex form. Furthermore, the release of TR from the 1:2 TR-HPβCD complex was slower when compared to the 1:1 complex. This may be due to the stronger hydrophobic interactions of TR with the 2 molecules of HPβCD resulting from the cage structure, formed by 2 molecules of HPβCD, which may hinder the escape of the TR molecule from the complex. Although the OC-HPβCD complex shows the same cage structure, the delay in release was not profound as in TR-HPβCD complex 1:2. This can be attributed to that the binding affinity of HPβCD to OC is lower than its binding affinity to TR and that the number of OC molecules in the complex is higher than the number of HPβCD molecules. Consequently, not all OC molecules are strongly entrapped within the cavity of the cage structure of the two HPβCD molecules, and can thus dissociate from the complex more easily. This assumption is supported by comparing the release profile of all the complexes studied (Fig. 5d). In all cases, higher number of HPβCD molecules in the complex is associated with a longer delay of the drug release therefrom.

As drug-CD complexes are known not to pass through the gastrointestinal barrier (35), the release studies were performed with a semipermeable membrane allowing the passage of the drugs, but not the CDs or the complexes. Given the instantaneous complex formation, the gastrointestinal absorbance of these drugs will be slower in the presence of CDs. As a result, the therapeutic efficacy, especially in case of the investigated opioid drugs, might be strongly affected. The water-soluble drugs were selected to demonstrate that complexes with CDs can be formed even after complete dissolution. Despite the complexes’ water solubility, they may delay the absorption of the drugs, as the rate of drug dissociation from CDs might be the speed limiting step. This becomes more relevant when considering that the gastric transit time is limited, and that a complete drug dissociation and release was in this case achieved in 48 h. In case of the drugs used as potent analgesics, such as TR and OC, time is a very important factor in increasing the quality of treatment. Accordingly, the concomitant administration of these drugs and other dosage forms containing CD as excipients may lead to an inadvertent delay in the desired analgesic effect. Therefore, the example presented here highlights the importance of an in-depth analysis of the potential drug-excipient interactions, which can be integrated as a screening step before or during the formulation development of pharmaceutical products.

## CONCLUSION

This study confirms the ability of CD to spontaneously form complexes with water-soluble drugs and the impact of such a complex formation on the drugs’ release behavior. ITC results and molecular modeling indicate that CD have higher binding affinity to drugs with higher hydrophobicity and structural flexibility. Additionally, complex formation was shown to retard the drug permeation across semipermeable membranes, as the drug first needs to dissociate from the complex. The rate of permeation was proportional to the ratio of CD molecules in the complex. Taken together, the current study highlights the potential of implementing a combination of ITC and molecular modeling for the detection of incompatibilities between water-soluble drugs and CD, which have been beyond serious considerations by now. Further studies are surely needed to detail whether this is a general formulation obstacle or remains limited in effect and to a small number of hydrophilic actives.

## Supplementary Information

Fig. S1 (DOCX 96 kb)
