# Supplementary figures and images for: Cyclodextrin Complex Formation with Water-Soluble Drugs: Conclusions from Isothermal Titration Calorimetry and Molecular Modeling
Source: AAPS PharmSciTech. 2021 Aug 31;22(7):232. doi: 10.1208/s12249-021-02040-8 (PMC8410728; doi:10.1208/s12249-021-02040-8)

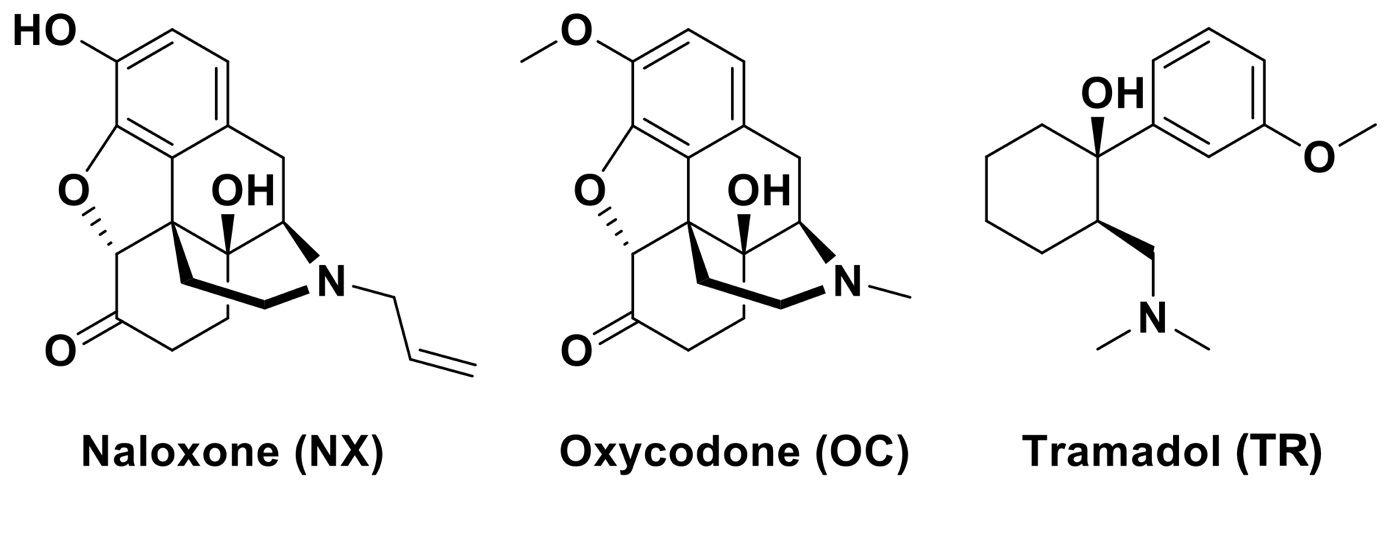


Figure S1: Chemical structure of the active ingredients

Supplement: Supplementary file 1 — (DOCX 96 kb) [file 12249_2021_2040_MOESM1_ESM.docx]
